# Quantitative evaluation of corneal irregularity and scarring after infectious keratitis using anterior segment optical coherence tomography

**DOI:** 10.1007/s00417-023-06157-3

**Published:** 2023-07-20

**Authors:** Kazunori Ichikawa, Takashi Ono, Lily Chen, Kohdai Kitamoto, Yukako Taketatni, Tetsuya Toyono, Junko Yoshida, Makoto Aihara, Takashi Miyai

**Affiliations:** 1 Department of Ophthalmology, The University of Tokyo Hospital, 7-3-1 Hongo, Bunkyo-Ku, Tokyo, 113-8655 Japan; 2 Department of Ophthalmology, Graduate School of Medicine, University of Tokyo, 7-3-1 Hongo, Bunkyo-Ku, Tokyo, Japan; 3 Department of Ophthalmology, International University of Health and Welfare Mita Hospital, 1-4-3 Mita, Minato-Ku, Tokyo, Japan

**Keywords:** Anterior segment optical coherence tomography, Corneal scarring, Infectious keratitis, Irregular corneal astigmatism

## Abstract

**Purpose:**

Corneal scars after infectious keratitis lead to insufficient transparency and irregular astigmatism, affecting visual acuity; therefore, they should be accurately evaluated to estimate visual function. This study aimed to quantitatively evaluate corneal irregularity and scarring after infectious keratitis using anterior segment optical coherence tomography (AS-OCT).

**Methods:**

This was an observational clinical study. We included patients who had corneal scarring after treatment of infectious keratitis between 2014 and 2021 at University of Tokyo Hospital. We retrospectively examined best spectacle-corrected visual acuity (BSCVA), average keratometric power, central corneal thickness (CCT), and four components of the Fourier harmonic analysis including spherical and asymmetry components, as well as regular astigmatism and higher-order irregularity. We included anterior and posterior corneal data and compared results with those of contralateral healthy eyes. Additionally, we quantitatively evaluated the densitometry of the cornea obtained using AS-OCT.

**Results:**

A total of 122 eyes of 61 patients were examined; male predominance was observed (n = 37), and the mean patient age was 55.3 ± 19.4 years. Comparisons with contralateral healthy eyes showed that BSCVA worsened (0.30 ± 0.83 and 0.93 ± 1.36 logMAR, respectively, *P* = 0.003), and CCT (531.1 ± 46.2 and 591.8 ± 132.4 μm, respectively, *P* < 0.001) and corneal densitometry (84.4 ± 11.8 and 111.9 ± 19.2 grayscale units, respectively, *P* < 0.001) increased significantly in affected eyes. The asymmetry component and higher-order irregularities that were not corrected with spectacles significantly increased (both* P* < 0.001), and there were no significant differences in the changes among the bacterial, fungal, herpetic, and acanthamoeba types of keratitis.

**Conclusion:**

Corneal scarring persisted after treatment for infectious keratitis, and the asymmetry and irregularities of corneal astigmatism increased as visual acuity deteriorated. AS-OCT with the Fourier harmonic analysis was useful for evaluating corneal topographic changes in patients with corneal scarring after keratitis.

**Supplementary Information:**

The online version contains supplementary material available at 10.1007/s00417-023-06157-3.


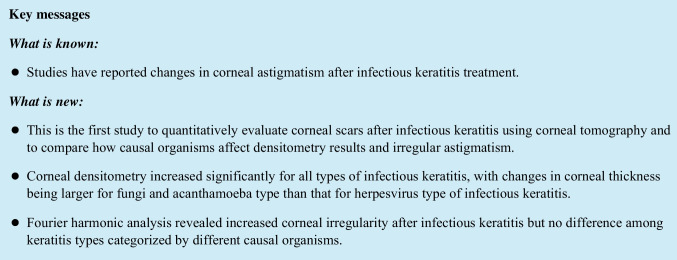


## Introduction

Infectious keratitis is a condition in which pathogens such as bacteria, viruses, and fungi cause corneal abscess formation and inflammation [1]. In some cases, corneal scarring can develop even with proper antibiotic and anti-inflammatory treatment, leading to long-term visual impairment and necessitating therapeutic or phototherapeutic corneal transplantation. Infectious keratitis is the third most common reason for corneal transplantation among primary diseases [2]. Corneal scarring, a leading cause of blindness worldwide, is caused primarily by infectious keratitis [3, 4].

Corneal astigmatism is an important factor to consider in the maintenance of visual acuity. It is observed not only in patients with corneal diseases but also in those without ophthalmological issues [5, 7]. Scarring after infectious keratitis can affect visual function due to the invasion of the visual axis [8] and increasing higher-order corneal aberrations [8, 12]. Corneal astigmatism has also been increasingly reported in patients with keratitis [1, 13], and previous studies have reported changes in higher-order aberrations of anterior and posterior surfaces after corneal diseases [14]. In our clinical experience, many patients with fungal keratitis have strong corneal opacity, while herpetic keratitis usually results in slight opacity that is localized in the corneal epithelium. This suggests that the degree of the opacity and corneal deformation can be different depending on the cause of keratitis. However, no studies have quantitatively evaluated corneal scars by comparing how different causes of infectious keratitis influenced irregular astigmatism measured with corneal tomography.

Thus, we aimed to evaluate the degree of corneal irregular astigmatism and opacification after infectious keratitis using anterior segment optical coherence tomography (AS-OCT).

## Methods

### Ethics consideration

This was a retrospective observational clinical study performed at a single tertiary hospital in Japan. This study was approved by the Institutional Review Board of the Research Ethics Committee of the University of Tokyo Hospital (Identifier: 2020006NI) and was conducted according to the tenets of the Declaration of Helsinki. The opt-out method of consent was used, wherein participants were given the option to opt-out of the clinical study. Requirement of written informed consent was waived in this process.

### Patients

Included patients were those who visited a specialized corneal clinic at University Tokyo Hospital and underwent corneal tomographic analysis for scarring after infectious keratitis caused by bacteria, fungi, herpesvirus, and acanthamoeba between January 2014 and May 2021. We excluded patients who developed a corneal infection after transplantation, underwent therapeutic corneal transplantation, and had another corneal disease or a history of corneal surgery. Corneal tomographic analysis was performed using AS-OCT (CASIA or CASIA2; Tomey Corporation, Inc., Aichi, Japan) within 1 year after complete recovery from abscess without any corneal epithelial defects.

### Examination items

We categorized patients into four groups based on the primary cause of keratitis: bacteria, fungi, herpesvirus, and acanthamoeba. We compared the corneal parameters of eyes with infectious keratitis after complete recovery of the epithelium (the affected eyes) with those of the contralateral eyes without ophthalmological disease (the healthy eyes). We also compared age, best spectacle-corrected visual acuity (BSCVA), average keratometry (AvgK), central corneal thickness (CCT), thinnest corneal thickness (TCT), and four components of the Fourier harmonic analysis of corneal data within the 6.0 mm diameter, including regular astigmatism, higher-order irregularity, and asymmetric and spherical components. Additionally, we quantitatively evaluated corneal density based on the image obtained using AS-OCT with equipment software. The values reflected the brightness of the corneal image within a dimeter of 4.0 mm and indicated the degree of opacity, where 0 (black) indicated slight scattering and 255 (white) indicated substantial scattering.

Fourier harmonic analysis of corneal topographic data was performed as previously reported [15, 16]. Anterior and posterior corneal refractive power data on the 3-mm Mire ring were decomposed into a series of trigonometric components. The dioptric powers on Mire ring I and Fi ($\upsigma$) values were transformed into trigonometric components using the Fourier series harmonic analysis program included in CASIA or CASIA2:

$${\mathrm{F}}_{\mathrm{i}}\left(\upsigma \right)={a}_{0}+{c}_{1}\mathrm{cos}\left(\sigma -{\alpha }_{1}\right)+{c}_{2}\mathrm{cos}2\left(\sigma -{\alpha }_{2}\right)+ {c}_{3}\mathrm{cos}3\left(\sigma -{\alpha }_{3}\right)+\dots +{c}_{n}\mathrm{cos}n\left(\sigma -{\alpha }_{n}\right)$$

where a_0_, 2c_1_, 2c_2_, and c_3_…_n_ represented the spherical, asymmetry (tilt or decentration), regular astigmatism, and higher-order irregularity components, respectively. These calculations were performed on rings 2–9, which approximated the central 6-mm zone of the cornea, and the results were averaged for each of the four parameters. All calculations were performed using CASIA or CASIA2 software. In the present study, we performed multiple AS-OCT examinations and included only the clinical data from examinations with good fixation and repeatability.

### Statistical analyses

Unless otherwise specified, the values are expressed as mean ± standard deviation. Visual acuity was converted to the logarithmic form of the minimum angle of resolution (logMAR) for calculation and analysis. A paired t-test was performed to compare the healthy and affected eyes. The ratio between the groups was compared using the Chi-square test. One-way analysis of variance with Tukey multiple comparison was performed to compare parameters among four groups of infectious keratitis. Analyses were performed using GraphPad Prism (GraphPad Software, San Diego, CA, USA), and statistical significance was set at *P* < 0.05.

## Results

**Fig. 1:**
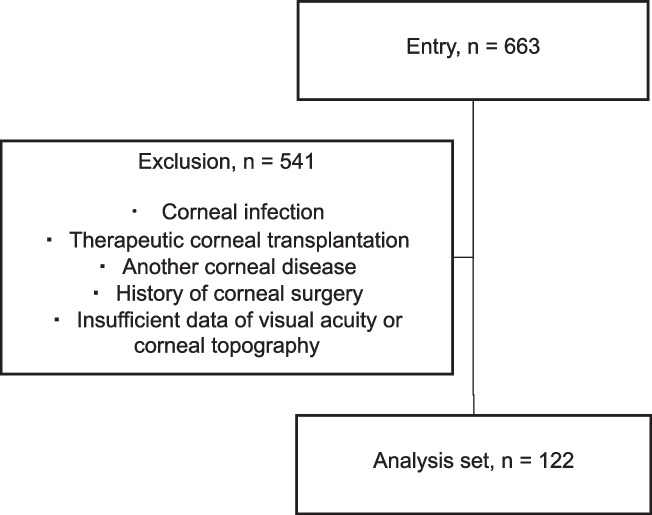
Flow diagram of the patients included in the study

The medical charts of 663 eyes were reviewed, and 541 eyes were excluded from the study because of insufficient information or a history of corneal surgery. A total of 61 patients and 122 eyes were included in the study (Fig. 1). The demographic data are summarized in Table 1. Male predominance was observed (n = 37), and the mean age of the patients was 55.3 ± 19.4 years. There was no significant difference in age or sex among the four groups categorized based on the primary causes of keratitis (bacteria, fungi, herpesvirus, and acanthamoeba). Degrees of haziness were negatively correlated with CCT for all patients (Y = -0.04199*X + 119.8, *P* = 0.033).

**Table 1:** Demographic data of patients with infectious keratitis scarring

	Total (eyes)	Bacteria (eyes)	Fungi (eyes)	Herpesvirus (eyes)	Acanthamoeba (eyes)
N (patients)	61	15	28	9	9
Total number of eyes	122	30	56	18	18
Affected eyes	61	15	28	9	9
Contralateral healthy eyes	61	15	28	9	9
Age (years)	58.3 ± 19.4	50.9 ± 22.4	61.6 ± 13.4	63.1 ± 21.0	55.6 ± 18.7
Sex ratio: Male/Female (patients)	37/24	11/4	16/12	4/5	6/3

**Fig. 2:**
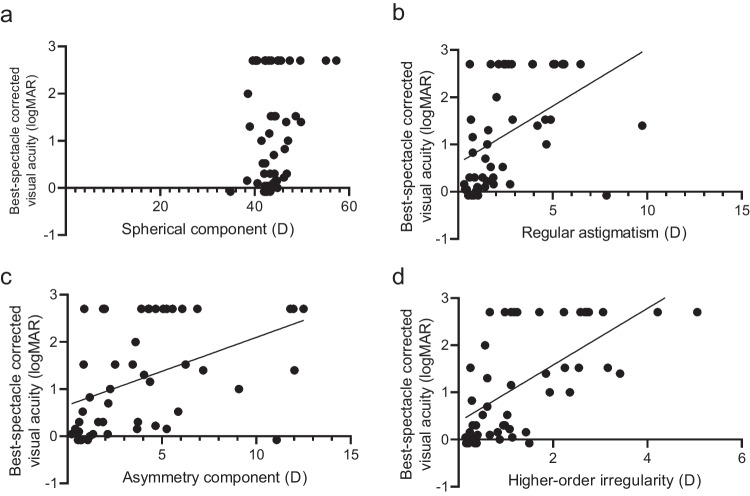
Comparison of averaged keratometry and corneal thickness between affected and contralateral healthy eyes**. a**. No significant difference was found among the four causes of infectious keratitis in difference of AvgK between affected and healthy eyes. **b**. A significant difference was observed among the four causes of infectious keratitis in difference of CCT between affected and healthy eyes (*P* = 0.04). **c**. A significant difference was observed among the four causes of infectious keratitis in difference of TCT between affected and healthy eyes (*P* = 0.02). It was higher in fungi and acanthamoeba than that in herpesvirus (*P* = 0.03 and *P* = 0.03, respectively). Analysis of variance with multiple comparisons was used. AvgK, averaged keratometry; CCT, central corneal thickness; TCT, thinnest corneal thickness

A comparison of the corneal data for all patients is summarized in Table 2. BSCVA was significantly worse in affected eyes compared with in healthy eyes (0.93 ± 1.36 and 0.30 ± 0.83 logMAR, *P* = 0.003). Although no difference was found in AvgK and TCT between the two groups, CCT was significantly higher in affected eyes compared with in healthy eyes (591.8 ± 132.4 and 531.1 ± 46.2 μm, *P* = 0.001). Haziness was also significantly worse in affected eyes (111.9 ± 19.2 and 84.4 ± 11.8 grayscale units (GSU), *P* < 0.001). The Fourier harmonic analysis revealed that asymmetry and higher-order (irregular corneal) astigmatism were significantly increased in affected eyes, similar to regular astigmatism (*P* < 0.001 for all). There were significant positive correlations between BSCVA and the three components of the Fourier harmonic analysis (regular astigmatism: R^2^ = 0.22, *P* < 0.001, irregular astigmatism: R^2^ = 0.20, *P* = 0.002, higher-order irregularity: R^2^ = 0.39, *P* < 0.001). There were no significant differences in changes in BSCVA and AvgK among the four types of keratitis (bacteria, fungi, herpes, and acanthamoeba) (Fig. 2A); however, changes in CCT were significantly different among the groups (40.6 ± 108.1, 106.0 ± 108.9, -5.6 ± 65.1, and 121.6 ± 131.6 D, respectively; *P* = 0.04) (Fig. 2B). Changes in TCT were also significantly different among the four groups (bacteria, fungi, herpes, and acanthamoeba: -15.7 ± 59.4, 20.0 ± 60.7, -74.0 ± 107.1, and 58.4 ± 136.9 μm, respectively; *P* = 0.015) (Fig. 2C), and multiple comparisons revealed that TCT changes after fungal and acanthamoeba keratitis were significantly greater than those after herpesvirus (*P* = 0.03 for both) (Fig. 2C).

**Table 2:** Comparison of corneal data of all patients included in the study

	Healthy eye	Affected eye	p-value
BSCVA (logMAR)	0.30 ± 0.83	0.93 ± 1.36	0.003
AvgK (D)	44.2 ± 2.2	46.1 ± 7.6	0.06
CCT (μm)	531.1 ± 46.2	591.8 ± 132.4	0.001
TCT (μm)	514.4 ± 41.8	497.0 ± 119.1	0.30
Densitometry (GSU)	84.4 ± 11.8	111.9 ± 19.2	< 0.001
Fourier harmonic analysis			
- Spherical component (D)	42.9 ± 2.2	44.6 ± 7.1	0.09
- Regular astigmatism (D)	1.1 ± 1.4	2.8 ± 2.3	< 0.001
- Asymmetry component (D)	1.4 ± 2.1	4.5 ± 4.2	< 0.001
- Higher-order astigmatism (D)	0.6 ± 1.1	1.5 ± 1.1	< 0.001

Affected and contralateral healthy eyes of each of the 61 patients in the present study were evaluated. BSCVA, best-spectacle-corrected visual acuity; logMAR, logarithm of the minimum angle of resolution; AvgK, averaged keratometry; CCT, central corneal thickness; TCT, thinnest corneal thickness; GSU, gray scale units

**Fig. 3:**
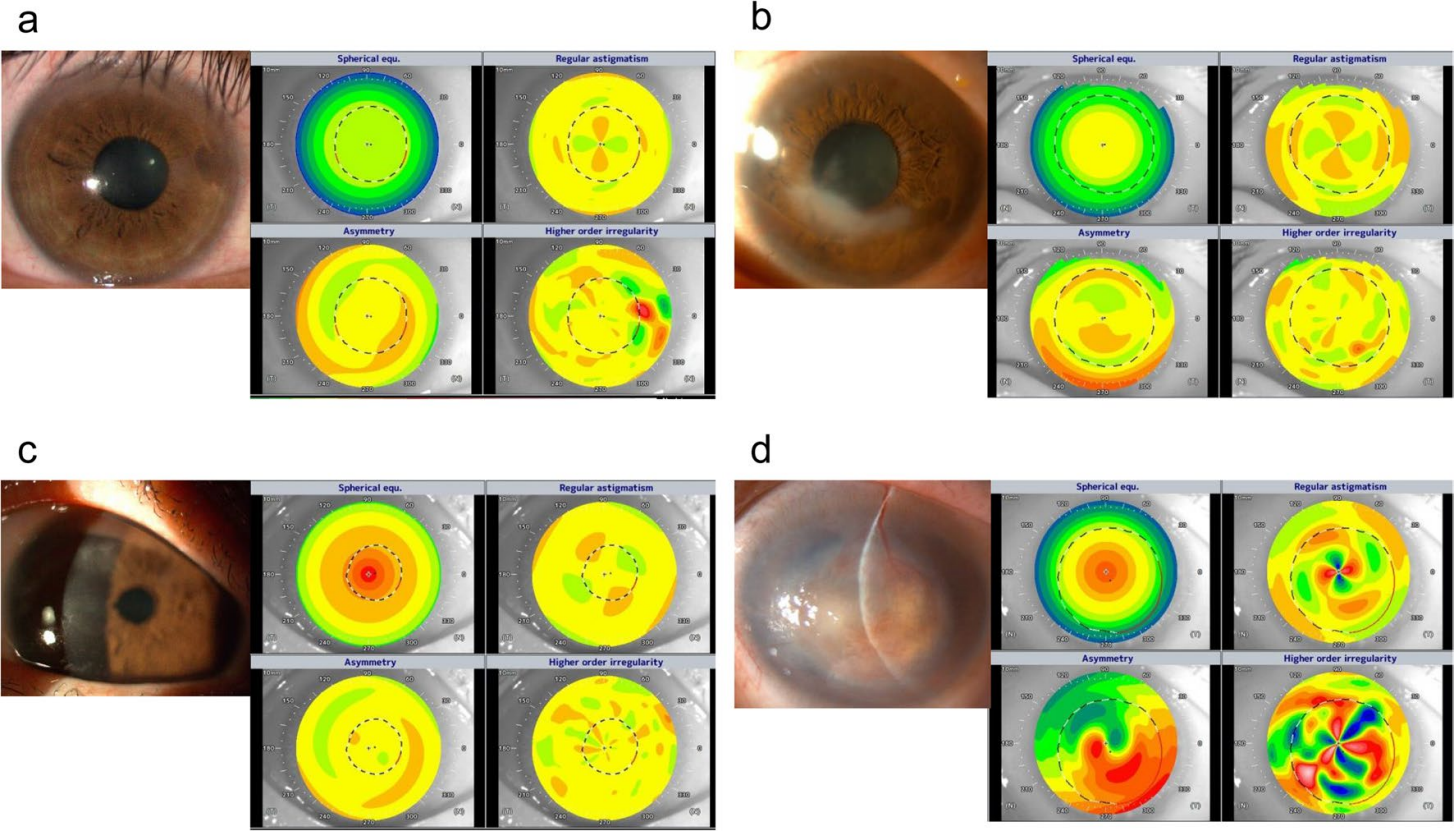
Representative images of anterior segment and the Fourier harmonic analysis of the cornea. **a**. Corneal scar after bacterial keratitis at the 3 o’clock position. **b**. Corneal scar after fungal keratitis at the 5 − 9 o’clock position. **c**. Corneal scar after herpetic keratitis at the 9 − 11 o’clock position. **d**. Corneal scar after Acanthamoeba keratitis at the central cornea

### Bacterial keratitis

Table 3 summarizes the corneal data of patients with scarring due to bacterial keratitis. More haziness (*P* = 0.001) and worse BSCVA (*P* = 0.007) were observed in affected eyes compared with in healthy eyes. The Fourier harmonic analysis revealed significantly increased regular astigmatism, asymmetry, and higher-order irregularity (*P* = 0.03, *P* = 0.04, and *P* = 0.002, respectively). Representative images are shown in Fig. 3A.

**Table 3:** Comparison of corneal data of scarring after the four types of infectious keratitis

		BSCVA (logMAR)	AvgK (D)	CCT (μm)	TCT (μm)	Densitometry (GSU)	Spherical component (D)	Regular astigmatism (D)	Asymmetry component (D)	Higher-order astigmatism (D)
Bacteria	Healthy eye	0.24 ± 0.73	44.7 ± 2.0	519.0 ± 45.6	507.2 ± 40.2	81.3 ± 8.4	43.5 ± 1.7	0.9 ± 0.7	1.0 ± 1.4	0.4 ± 0.5
	Affected eye	1.02 ± 1.09	46.0 ± 4.3	567.9 ± 107.9	492.1 ± 75.9	108.1 ± 22.1	44.8 ± 4.3	2.2 ± 1.9	2.4 ± 1.8	1.1 ± 0.8
	P-value	0.007	0.32	0.14	0.53	0.001	0.32	0.03	0.04	0.002
Fungi	Healthy eye	0.24 ± 0.74	43.2 ± 2.1	527.5 ± 43.0	513.9 ± 44.4	86.0 ± 14.2	41.9 ± 2.3	1.3 ± 1.7	2.0 ± 2.8	1.0 ± 1.6
	Affected eye	1.27 ± 1.07	44.2 ± 3.4	627.6 ± 103.6	530.3 ± 79.4	111.4 ± 17.8	42.9 ± 3.3	2.6 ± 2.0	4.2 ± 3.6	1.4 ± 1.1
	P-value	< 0.001	0.2	< 0.001	0.36	< 0.001	0.33	0.03	0.0497	0.23
Herpesvirus	Healthy eye	0.88 ± 1.29	45.5 ± 1.7	556.5 ± 49.8	523.1 ± 35.6	86.3 ± 13.2	44.3 ± 1.6	1.3 ± 1.7	0.7 ± 0.7	0.4 ± 0.4
	Affected eye	0.86 ± 0.81	46.9 ± 4.9	546.0 ± 42.4	438.8 ± 123.9	107.0 ± 17.7	46.8 ± 4.1	3.1 ± 3.3	4.3 ± 4.8	1.6 ± 1.8
	P-value	0.97	0.43	0.64	0.08	0.01	0.04	0.03	0.052	0.06
Acanthamoeba	Healthy eye	0.06 ± 0.23	44.6 ± 2.5	534.3 ± 52.2	516.6 ± 49.2	82.3 ± 5.7	42.9 ± 2.2	0.5 ± 0.3	0.7 ± 0.7	0.2 ± 0.04
	Affected eye	2.00 ± 0.85	51.2 ± 17.1	563.2 ± 252.2	459.3 ± 221.9	126.9 ± 16.0	42.5 ± 3.0	2.5 ± 1.3	5.3 ± 2.7	1.6 ± 1.0
	P-value	< 0.001	0.29	0.75	0.47	< 0.001	0.77	0.01	0.01	0.02

Affected and contralateral healthy eyes of each of the 61 patients in the present study were evaluated. BSCVA, best-spectacle-corrected visual acuity; logMAR, logarithm of the minimum angle of resolution; AvgK, averaged keratometry; CCT, central corneal thickness; TCT, thinnest corneal thickness.; GSU, gray scale units; D, diopters

### Fungal keratitis

Table 3 demonstrates that more haziness and worse BSCVA were observed in affected eyes compared with in healthy eyes (*P* < 0.001 for both). Additionally, the CCT of affected eyes was significantly higher (*P* < 0.001). The Fourier harmonic analysis revealed significantly increased regular astigmatism and asymmetry components (*P* = 0.03 and 0.0497, respectively). Representative images are shown in Fig. 3B.

### Herpetic keratitis

The corneal data of patients with scarring due to herpetic keratitis showed that although haziness was significantly worse (*P* = 0.01), no change was observed in the BSCVA of affected eyes compared with in healthy eyes. The Fourier harmonic analysis revealed significantly increased spherical components and regular astigmatism (*P* = 0.04 and *P* = 0.03, respectively). Representative images are shown in Fig. 3C.

### Acanthamoeba keratitis

Corneal data of patients with scarring due to acanthamoeba keratitis showed more haziness (*P* < 0.001) and worse BSCVA (*P* < 0.001) in affected eyes compared with in healthy eyes. The Fourier harmonic analysis revealed significantly increased regular astigmatism, asymmetry, and higher-order irregularities (*P* = 0.01, *P* = 0.01, and *P* = 0.02, respectively). Representative images are shown in Fig. 3D.

We analyzed the differences in each parameter of the Fourier harmonic analysis based on the causative organism of infectious keratitis. No significant differences were found in the anterior (Supplemental Fig. 1) or posterior (Supplemental Fig. 2) cornea.

## Discussion

We demonstrated that corneal scarring after bacterial, fungal, and acanthamoeba keratitis results in significant visual impairment. Shimizu et al. [17] reported visual acuity after bacterial (0.98 ± 1.01), fungal (1.30 ± 1.17), herpetic (1.08 ± 0.95), and acanthamoeba (0.60 ± 0.91) keratitis. In the present study, BSCVA approximately coincided with these values; however, it was more severe in patients with acanthamoeba keratitis (2.00 ± 0.85). This could be because our center is a tertiary hospital in the inner-city area, where more advanced cases are often referred. Although there was no difference in AvgK in all patients with keratometry values, the Fourier harmonic analysis detected some changes in corneal tomography findings. Our results demonstrated that regular astigmatism, asymmetry components, and higher-order irregularities significantly increased in eyes with infectious keratitis. Since all these factors were significantly related to BSCVA, these three Fourier harmonic analysis parameters are thought to contribute to the maintenance of visual acuity. The asymmetry component and higher-order irregularity cannot be corrected with glasses and require rigid gas-permeable lenses for correction. The results suggest that many patients with corneal scarring need treatment for visual correction even after recovery from infectious keratitis. The AS-OCT in our study was useful for evaluating corneal parameters [18, 19]. Scheimpflug imaging is another modality that is useful for evaluating corneal scars with sufficient reproducibility and repeatability [20]. However, it may underestimate corneal thickness in the presence of central scarring [21]. Therefore, AS-OCT is considered more suitable for evaluating hazy corneas with post-infectious scars.

Anterior and posterior corneal topographic changes were evaluated in our study. The posterior curvature of the cornea, which cannot be treated with a rigid gas-permeable lens, is important for visual function [22]. Although Fourier harmonic components were not different among four causative organisms of infectious keratitis, a high value of asymmetry component was observed in all patients. Therefore, a detailed evaluation of posterior corneal astigmatism for scarring is necessary even after complete recovery. Additionally, after the treatment of infectious keratitis, fabrication of eyeglasses or ophthalmic surgery, such as cataract surgery, require accurate corneal astigmatism analysis, especially for patients with a toric lens inserted for astigmatism correction [23].

We evaluated corneal scarring using densitometry of the images obtained from AS-OCT. Affected eyes exhibited higher values in all four types of infectious keratitis categorized by causal organisms. Corneal densitometry has been shown to be useful for quantitatively evaluating opacity by AS-OCT [24], as well as Scheimpflug imaging [25, 26]. The degree of opacity was traditionally evaluated with slit-lamp microscopic examination, but it was difficult to measure and compare the earlier data of the same patient with that of a different one. Densitometry of AS-OCT can quantitatively evaluate corneal scarring, and it is useful for not only patient-to-patient comparison but also longitudinal comparison of the data of the same patients.

We found that CCT increased in patients after fungal and acanthamoeba keratitis, in contrast to those with bacterial and herpetic keratitis, where no changes were observed. Fungal keratitis can deeply invade the corneal stroma and cause severe persistent inflammation that sometimes requires intrastromal injection of antifungal medications [27]. Additionally, topical medications for fungi, such as chlorhexidine, can cause toxicity to the cornea. Higher-order aberrations have been reported to be prevalent in fungal keratitis [17]. Although herpetic keratitis could affect both the corneal epithelial and stromal layers, where the virus was detected at different depths [28], stromal keratitis accounted for only 29.5% of cases, where superficial keratitis was the main cause [29, 30]. These differences in the depth of the infectious foci could explain the results of our study. Furthermore, densitometry values significantly increased in patients with herpetic keratitis, although CCT did not change. This suggests that the solid corneal opacity remained without thickening even after treatment for keratitis. There was variability in the formation of corneal scars after herpetic keratitis and acanthamoeba keratitis. It should be noted that our study group of patients with herpetic keratitis did not have severe stages of the disease. Patients with herpetic keratitis who are unresponsive to treatment can demonstrate progressive corneal opacity. However, as our institution is a tertiary care hospital located in the central area of the capital city in Japan, patients were typically not left untreated for a long time. This particularly geographical characteristic is a distinctive feature of the patients included in the study.

AS-OCT is useful for understanding corneal astigmatism owing to corneal scarring after treatment for infectious keratitis because the type of persisting corneal astigmatism or corneal haziness is tightly linked to the post-keratitis options for visual correction, such as the use of optical glasses or RGP lens. Patients with mild corneal haziness and high corneal irregular astigmatism can be prescribed RGP lens for correction.

This study has several limitations. First, we compared the affected eyes after keratitis with the contralateral healthy eyes. This is because most of the included patients had not visited our center before they developed keratitis; thus, corneal data and BSCVA of the affected eyes before infectious events were unavailable. Second, it would have been beneficial to factor in the strain of the organism when evaluating corneal tomographic changes and group patients based on specific etiologies such as gram-positive bacteria, gram-negative bacteria, Candida, and Aspergillus. However, this was difficult because of the limited number of patients with sufficient corneal data. Furthermore, in the present study, the number of eyes with herpetic keratitis and acanthamoeba keratitis were small. Further prospective studies including large numbers of eyes, for each of the etiology groups, are required in the future to validate the findings. Lastly, although we observed a statistical difference in corneal thickness in some analyses, the value was not high. Consequently, we had to verify the repeatability of the examination results because of the low visual acuity and insufficient fixation at times. Herein, we performed multiple examinations and adopted only reliable clinical data.

In conclusion, corneal scarring persisted even after treatment for infectious keratitis, and regular and irregular corneal astigmatism increased with visual acuity impairment. AS-OCT with the Fourier harmonic analysis was useful for evaluating corneal topographic changes in patients with corneal scarring after keratitis. Future multi-institutional studies that include data before and after keratitis may be helpful for clarifying how AS-OCT with Fourier harmonic analysis can be used in a clinical setting.

### Supplementary Information

Below is the link to the electronic supplementary material.

Supplementary file1 (DOCX 28 KB)

Supplementary file2 (PPTX 141 KB)

Supplementary file3 (PPTX 140 KB)

## Data Availability

The data that support the findings of this study are available from the corresponding author upon request. The data are not publicly available because they contain information that may compromise the privacy of research participants.

## References

[1] Tuli SS, Schultz GS, Downer DM (2007). Science and strategy for preventing and managing corneal ulceration. Ocul Surf.

[2] Gain P, Jullienne R, He Z (2016). Global survey of corneal transplantation and eye banking. JAMA Ophthalmol.

[3] Austin A, Lietman T, Rose-Nussbaumer J (2017). Update on the management of infectious keratitis. Ophthalmology.

[4] Pascolini D, Mariotti SP (2012). Global estimates of visual impairment: 2010. Br J Ophthalmol.

[5] Liu Z, Huang AJ, Pflugfelder SC (1999). Evaluation of corneal thickness and topography in normal eyes using the Orbscan corneal topography system. Br J Ophthalmol.

[6] Ueno Y, Nomura R, Hiraoka T, Kinoshita K, Ohara M, Oshika T (2021). Comparison of corneal irregular astigmatism by the type of corneal regular astigmatism. Sci Rep.

[7] Reddy SP, Bansal R, Vaddavalli PK (2013). Corneal topography and corneal thickness in children. J Pediatr Ophthalmol Strabismus.

[8] Menda SA, Das M, Panigrahi A (2020). Association of postfungal keratitis corneal scar features with visual acuity. JAMA Ophthalmol.

[9] Jiménez JR, Ortiz C, Pérez-Ocón F, Jiménez R (2009). Optical image quality and visual performance for patients with keratitis. Cornea.

[10] Kaswin G, Rousseau A, M'Garrech M (2013). Optical aberrations in patients with recurrent herpes simplex keratitis and apparently normal vision. Br J Ophthalmol.

[11] Shimizu E, Yamaguchi T, Tsubota K, Shimazaki J (2019). Corneal higher-order aberrations in eyes with corneal scar after traumatic perforation. Eye Contact Lens.

[12] Kashizuka E, Yamaguchi T, Yaguchi Y, Satake Y, Shimazaki J (2016). Corneal higher-order aberrations in herpes simplex keratitis. Cornea.

[13] Yoshihara M, Maeda N, Soma T (2015). Corneal topographic analysis of patients with Mooren ulcer using 3-dimensional anterior segment optical coherence tomography. Cornea.

[14] Yamaguchi T, Shimizu E, Yagi-Yaguchi Y, Tomida D, Satake Y, Shimazaki J (2017). A novel entity of corneal diseases with irregular posterior corneal surfaces: Concept and clinical relevance. Cornea.

[15] Oshika T, Tomidokoro A, Maruo K, Tokunaga T, Miyata N (1998). Quantitative evaluation of irregular astigmatism by Fourier series harmonic analysis of videokeratography data. Invest Ophthalmol Vis Sci.

[16] Ono T, Mori Y, Nejima R (2020). Long-term changes and effect of pterygium size on corneal topographic irregularity after recurrent pterygium surgery. Sci Rep.

[17] Shimizu E, Yamaguchi T, Yagi-Yaguchi Y (2017). Corneal higher-order aberrations in infectious keratitis. Am J Ophthalmol.

[18] Swartz T, Marten L, Wang M (2007). Measuring the cornea: The latest developments in corneal topography. Curr Opin Ophthalmol.

[19] Majander AS, Lindahl PM, Vasara LK, Krootila K (2012). Anterior segment optical coherence tomography in congenital corneal opacities. Ophthalmology.

[20] Das M, Menda SA, Panigrahi AK (2019). Repeatability and reproducibility of slit lamp, optical coherence tomography, and scheimpflug measurements of corneal scars. Ophthalmic Epidemiol.

[21] Khurana RN, Li Y, Tang M, Lai MM, Huang D (2007). High-speed optical coherence tomography of corneal opacities. Ophthalmology.

[22] Mohammadi SF, Khorrami-Nejad M, Hamidirad M (2019). Posterior corneal astigmatism: A review article. Clin Optom (Auckl).

[23] Lu LW, Rocha-de-Lossada C, Rachwani-Anil R, Flikier S, Flikier D (2021). The role of posterior corneal power in 21st century biometry: A review. J Fr Ophtalmol.

[24] Wang XY, Zhang TQ, Rachwani AR (2022). New algorithm for corneal densitometry assessment based on anterior segment optical coherence tomography. Eye (Lond).

[25] Tekin K, Kiziltoprak H, Koc M, Goker YS, Kocer AM, Yilmazbas P (2019). The effect of corneal infiltrates on densitometry and higher-order aberrations. Clin Exp Optom.

[26] Ní Dhubhghaill S, Rozema JJ, Jongenelen S, Hidalgo IR, Zakaria N, Tassignon MJ (2014). Normative values for corneal densitometry analysis by Scheimpflug optical assessment. Invest Ophthalmol Vis Sci.

[27] Donovan C, Arenas E, Ayyala RS, Margo CE, Espana EM (2022). Fungal keratitis: Mechanisms of Infection and Management Strategies. Surv Ophthalmol.

[28] Zhao G, Chen M, Liu T, Sun SY, Zhao J, Xie LX (2012). Association of HSV-1 antigen distribution in the cornea with clinical characteristics of herpetic stromal keratitis. Eur J Ophthalmol.

[29] Stuart-Harris C (1983). The epidemiology and clinical presentation of herpes virus infections. J Antimicrob Chemother.

[30] Labetoulle M, Auquier P, Conrad H (2005). Incidence of herpes simplex virus keratitis in France. Ophthalmology.

