# Supplementary material for: Quantitative evaluation of corneal irregularity and scarring after infectious keratitis using anterior segment optical coherence tomography
Source: Graefes Arch Clin Exp Ophthalmol. 2023 Jul 20;262(1):133–41. doi: 10.1007/s00417-023-06157-3 (PMC10805989; doi:10.1007/s00417-023-06157-3)
Supplement: Supplementary file 1 — Supplementary file1 (DOCX 28 KB) [file 417_2023_6157_MOESM1_ESM.docx]

**Supplemental Fig. 1** Comparison among the four components of the Fourier harmonic analysis of the anterior cornea

a. Changes in the spherical components after infectious keratitis.

b. Changes in regular astigmatism after infectious keratitis.

c. Changes in the asymmetry components after infectious keratitis.

d. Changes in higher-order irregularity after infectious keratitis.

There were no significant differences in each component of the Fourier harmonic analysis based on the causative organism of infectious keratitis.

**Supplemental Fig. 2** Comparison among the four components of the Fourier harmonic analysis of the posterior cornea

a. Changes in spherical components after infectious keratitis.

b. Changes in regular astigmatism after infectious keratitis.

c. Changes in the asymmetry components after infectious keratitis.

d. Changes in higher-order irregularity after infectious keratitis.

There were no significant differences in each component of the Fourier harmonic analysis based on the causative organism of infectious keratitis.
