# Supplementary figures and images for: Quantitative evaluation of corneal irregularity and scarring after infectious keratitis using anterior segment optical coherence tomography
Source: Graefes Arch Clin Exp Ophthalmol. 2023 Jul 20;262(1):133–41. doi: 10.1007/s00417-023-06157-3 (PMC10805989; doi:10.1007/s00417-023-06157-3)

## Slide 1
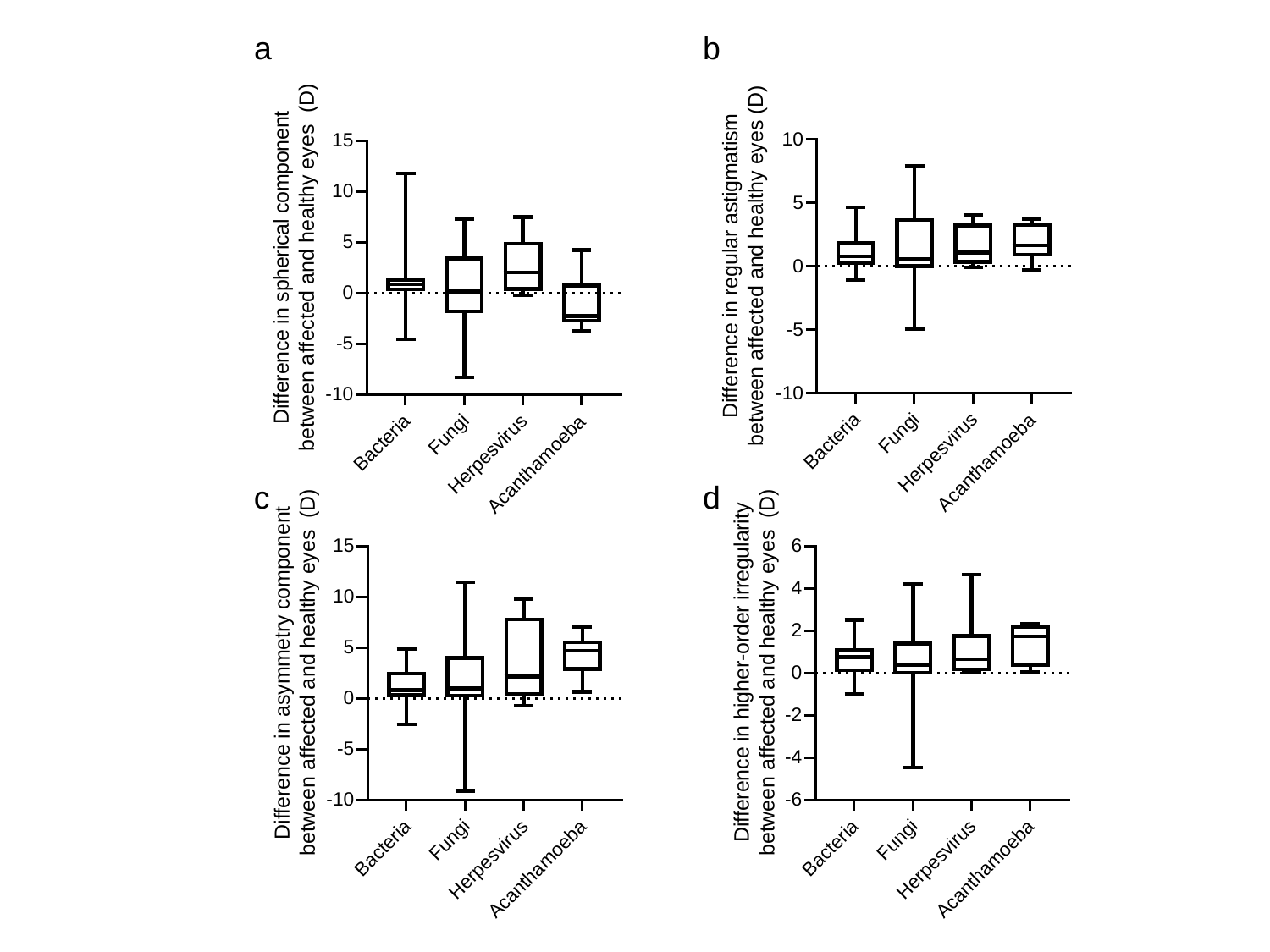

a
b
c
d

Supplement: Supplementary file 2 — Supplementary file2 (PPTX 141 KB) [file 417_2023_6157_MOESM2_ESM.pptx]

## Slide 1
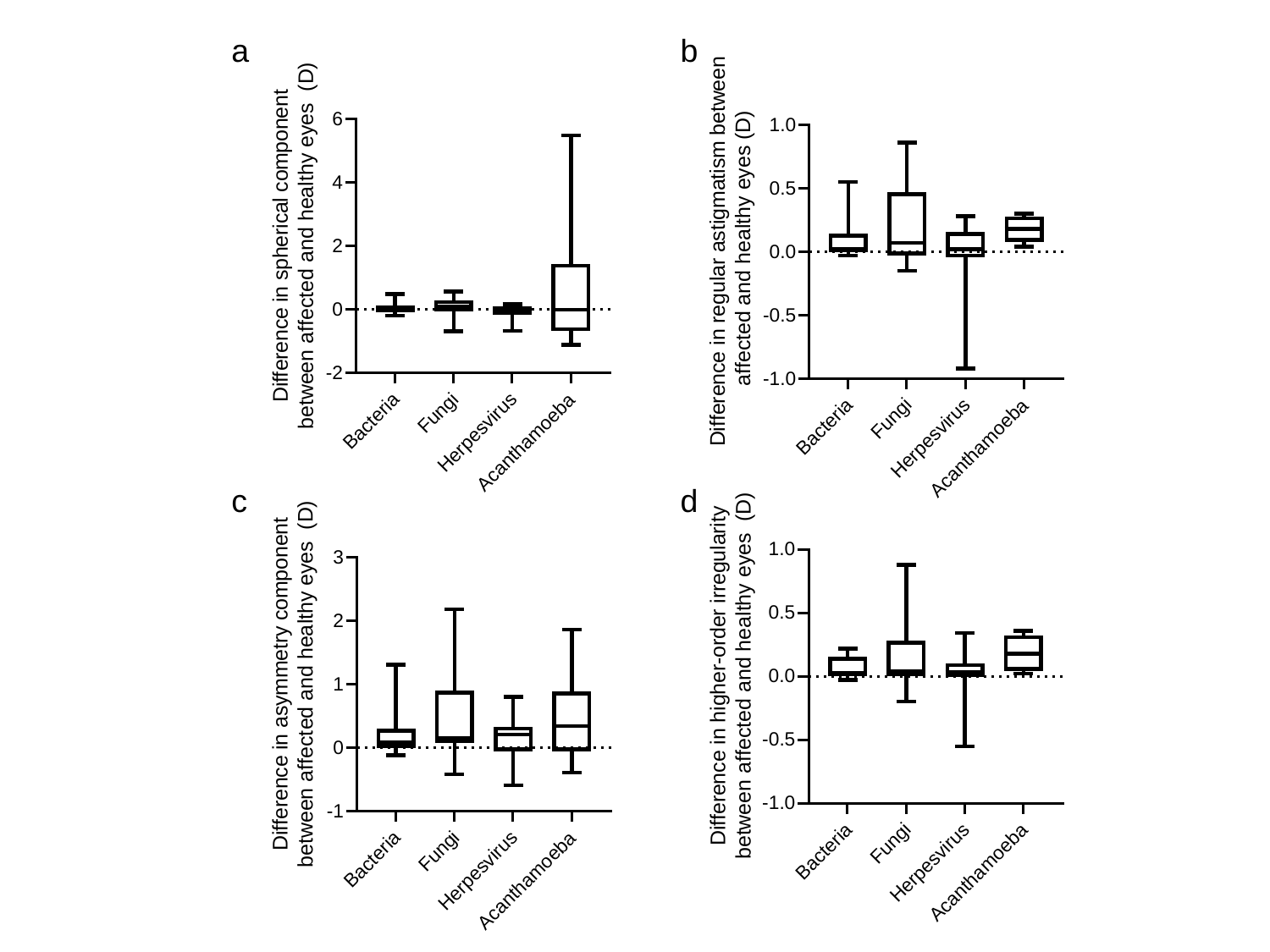

a
b
c
d

Supplement: Supplementary file 3 — Supplementary file3 (PPTX 140 KB) [file 417_2023_6157_MOESM3_ESM.pptx]
